# Factors associated with cognitive developmental delay risk in 12-month-old infants

**DOI:** 10.3389/fped.2026.1780613

**Published:** 2026-06-22

**Authors:** Yanping Zhang, Wei Li, Xiaochun He, Xiu Wang, Haoyue Gao, Jing Zeng

**Affiliations:** 1 School of Nursing, Chengdu Medical College, Chengdu, Sichuan, China; 2 Department of Child Health Care, Sichuan Provincial Maternity and Child Health Care Hospital, Chengdu, Sichuan, China; 3 Department of Nursing, Sichuan Provincial Maternity and Child Health Care Hospital, Chengdu, Sichuan, China

**Keywords:** 12-month-old infants, cesarean delivery, cognitive developmental delay risk, maternal obesity, socioeconomic status

## Abstract

**Objective:**

To identify factors associated with the risk of cognitive developmental delay in 12-month-old infants and provide evidence for early risk screening and intervention.

**Methods:**

A case–control study was conducted among 762 twelve-month-old children who underwent developmental screening with the Denver Developmental Screening Test (DDST) at Sichuan Provincial Maternity and Child Health Care Hospital from 2024 to 2025. Of these, 381 with suspected or abnormal development were assigned to the case group, and 381 with normal results served as controls. Perinatal, family, and caregiving factors were collected and were analyzed using univariate and multivariate logistic regression.

**Results:**

Cesarean delivery (OR = 1.42, 95% CI: 1.05–1.93), maternal alcohol use during pregnancy (OR = 9.12, 95% CI: 1.09–76.33), pre-pregnancy overweight (OR = 1.62, 95% CI: 1.02–2.58) and obesity (OR = 2.54, 95% CI: 1.08–5.95), inadequate (OR = 4.09, 95% CI: 1.74–9.62) or excessive (OR = 1.59, 95% CI: 1.10–2.29) gestational weight gain, and grandparental caregiving (OR = 1.80, 95% CI: 1.32–2.46) were independent risk factors. Higher paternal education (postgraduate or above; OR = 0.06, 95% CI: 0.01–0.56) and annual household income > 300,000 CNY (OR = 0.31, 95% CI: 0.12–0.80) were protective.

**Conclusion:**

Cognitive developmental risk at 12 months was associated with multiple perinatal, familial, and caregiving-related factors. A comprehensive assessment of potentially modifiable factors, including delivery mode, maternal weight status, gestational weight gain, prenatal alcohol exposure, and caregiving arrangements, may help identify infants at increased developmental risk and provide evidence for targeted early prevention and intervention strategies.

## 1 Introduction

Early neuropsychological development forms the foundation for lifelong mental health and social adaptation. Among its dimensions, cognitive development represents a central domain, intricately linked to basic functions such as language, attention, and memory, as well as to higher-order functions like emotional regulation and social cognition. A growing body of evidence suggests that cognitive levels during infancy and early childhood are closely associated with later learning capacity, social competence, and long-term health outcomes. Delayed cognitive development has been correlated with academic difficulties, impaired interpersonal relationships, an increased risk of mental health problems, and even chronic diseases in adulthood (1, 2). Therefore, early detection and assessment of cognitive developmental risks are crucial for promoting optimal long-term growth and wellbeing. The first year of life constitutes a critical window for rapid structural and functional remodeling of the brain. During this period, extensive synaptogenesis—the formation and strengthening of synaptic connections—drives the maturation of neural circuits. The temporal sequence of synapse formation across different brain regions ensures the staged and progressive nature of functional development. According to Gilmore et al. (3), gray matter growth in the first year of life progresses from posterior regions toward anterior regions, providing the neuroanatomical basis for early sensory-motor development and subsequent acquisition of language and higher-order cognition. By approximately 12 months of age, infants enter a critical phase of accelerated development in perception, motor control, cognition, language, and social interaction (4). Consequently, one year of age represents an optimal time window for identifying developmental risks and initiating early interventions. Among the available screening tools, the Denver Developmental Screening Test (DDST), which is characterized by simplicity, comprehensiveness, and broad applicability, remains one of the most widely used instruments for routine developmental screening in infants and young children (5).

Early cognitive development results from the interplay of genetic predisposition, biological conditions, family environment, and socioeconomic context. While genetic factors may define the potential range of cognitive capacity, environmental influences including maternal health and nutrition during pregnancy, delivery mode, parenting practices, socioeconomic status, and caregiving arrangements may play important moderating roles (6). Recent systematic reviews and meta-analyses have provided a broader evidence base for elucidating these relationships. A systematic review and meta-analysis of observational studies indicated that cesarean delivery is associated with a higher risk of neurodevelopmental and mental health disorders in offspring (7). Likewise, meta-analytic evidence demonstrates that maternal pre-pregnancy overweight and obesity are associated with elevated risks of adverse neurodevelopmental outcomes in children, including cognitive developmental delay (8). In addition, both inadequate and excessive gestational weight gain have been linked to unfavorable neurodevelopmental outcomes in offspring (9). Family-level factors may further modify or buffer these developmental risks. Although much of the available systematic review evidence comes from studies of children exposed to early biological risks such as preterm birth, these findings support a broader bio-psycho-social framework for understanding cognitive development (10, 11). Within this framework, socioeconomic conditions, parental education, caregiving quality, and family support may act as either risk-amplifying or protective factors. This perspective is particularly relevant to infancy, as early cognitive development depends not only on prenatal and perinatal conditions but also on postnatal stimulation, caregiver responsiveness, and access to early learning resources. However, inconsistencies remain across studies, particularly regarding the associations of cesarean delivery, maternal weight status, and gestational weight gain with child cognitive outcomes. Moreover, studies focusing on this critical early stage particularly at 12 months of age remain limited. Therefore, the present study aimed to examine factors associated with cognitive developmental delay among 12-month-old children, using DDST data collected at Sichuan Provincial Maternity and Child Health Care Hospital between 2024 and 2025. By focusing on this critical 12-month developmental window and comprehensively assessing modifiable perinatal, familial, and caregiving-related factors, this study may help clarify relatively stable epidemiological associations with early developmental risk and provide evidence for the development of targeted early intervention strategies.

## 2 Materials and methods

### 2.1 Participants

This case-control study was conducted at the Child Health Outpatient Department of Sichuan Provincial Maternal and Child Health Care Hospital between 2024 and 2025. A total of 762 children aged 12 months who underwent DDST assessments were included. The case group consisted of 381 children with “suspected/abnormal” developmental outcomes, and the control group included 381 children with “normal” results. Controls were randomly selected from DDST-negative children attending the same clinic during the study period. To minimize confounding, only full-term, singleton infants with normal birth weight, with no history of birth asphyxia or serious physical or neurological disorders were enrolled. In addition, all included children had undergone routine neonatal screening, including hearing screening at birth, and no abnormalities were reported. “Suspected” and “abnormal” categories were combined to represent screening-positive developmental risk and to improve sensitivity for early risk identification. This restriction helped reduce confounding by major medical conditions, allowing a clearer assessment of associations with perinatal, familial, and environmental factors.

### 2.2 Methods

#### 2.2.1 Data collection

Baseline data were obtained using a structured questionnaire designed by the research team based on prior studies and the aims of the current research. The collected information included: Child characteristics: age, sex, birth weight, and mode of delivery (cesarean or vaginal). Maternal characteristics: maternal age, pre-pregnancy BMI, gestational weight gain, pregnancy complications (gestational diabetes, hypertension), and breastfeeding history. Family and environmental factors: parental education and occupation, annual household income, primary caregiver, and type of residence (urban, suburban, or rural).

#### 2.2.2 Cognitive development screening

The DDST assessed cognitive developmental risk, first introduced in 1967 and widely used for developmental screening in children aged 0–6 years (12). The DDST is a standardized screening tool rather than a definitive diagnostic instrument; it assesses four domains: personal-social, fine motor–adaptive, language, and gross motor skills, encompassing 105 items. Testing requires approximately 10–20 min and is administered primarily through direct observation by the examiner, with a small number of items supplemented by caregiver reports when necessary. Results were categorized as normal, suspect, or abnormal according to standardized criteria: children were defined as abnormal if they met either of the following conditions: ① two or more domains each had two or more developmental delays; ② one domain had two or more developmental delays, plus one or more domains had one developmental delay with all items within the corresponding age line failed. Children were defined as suspect if they met either of the following conditions: ① one domain had two or more developmental delays; ② one or more domains had one developmental delay with all items within the corresponding age line failed. Children who did not meet the criteria for abnormal or suspect were classified as normal (13).

#### 2.2.3 Statistical analysis

Categorical variables were described using frequencies and percentages. Group differences were analyzed using the chi-square test or Fisher's exact test when appropriate. Variables with *P* < 0.05 in univariate analysis were entered into multivariate logistic regression models to identify independent risk factors. Statistical significance was defined as *P* < 0.05.

## 3 Results

### 3.1 General characteristics

A total of 762 one-year-old children were included in the analysis. Among them, 51.1% (*n* = 389) were delivered by cesarean delivery, 85.9% (*n* = 655) were first-born, and 47.0% (*n* = 358) were exclusively breastfed within the first 6 months. Appropriate gestational weight gain was observed in 71.9% (*n* = 548), and 81.0% (*n* = 617) of mothers had a pre-pregnancy BMI within the normal range (18.5–23.9 kg/m^2^). The prevalence of gestational diabetes and hypertension was 15.9% (*n* = 121) and 3.4% (*n* = 26), respectively. Most mothers reported no smoking (98.2%) or alcohol use (98.6%) during pregnancy. Parental age was primarily 30–34 years (fathers 45.7%, mothers 42.5%), and 77.6% (*n* = 591) of families were extended households. Male children accounted for 53.9% (*n* = 411). The primary caregiver was a grandparent in 54.1% (*n* = 412) of cases. Urban residents comprised 85.4% (*n* = 651), and most parents were engaged in mental rather than manual occupations (mothers 78.1%, fathers 63.0%). The majority of mothers (81.2%) and fathers (76.2%) held college or undergraduate degrees. Annual household income ranged mostly between 80,001 and 150,000 CNY (47.1%, *n* = 359).

### 3.2 Univariate analysis

Univariate analysis showed significant differences between the case and control groups in mode of delivery, maternal alcohol use during pregnancy, pre-pregnancy BMI, gestational weight gain, primary caregiver, paternal education, and household income (*P* < 0.05). Detailed results are presented in Table 1.

**Table 1:** Baseline characteristics by risk of cognitive developmental delay [n(%)].

Variable	Category	DDST Screening Results at 12 Months		Total	*χ*2	*P*
		Case group (*n* = 381)	Control group (*n* = 381)			
Parity	First birth	321 (84.3)	334 (87.6)	655		
	Second birth	60 (15.7)	45 (11.8)	105	-	0.099*
	Third birth	0 (0.0)	2 (0.6)	2		
Sex	Male	219 (57.5)	192 (50.4)	411	3.851	0.050
	Female	162 (42.5)	189 (49.6)	351		
Mode of delivery	Vaginal	172 (45.1)	201 (52.8)	373	4.417	0.036
	Cesarean delivery	209 (54.9)	180 (47.2)	389		
Feeding method within 6 months	Exclusive breastfeeding	175 (45.9)	183 (48.0)	358	1.351	0.509
	Mixed feeding	191 (50.1)	178 (46.7)	369		
	Formula feeding	15 (4.0)	20 (5.3)	35		
Gestational diabetes	No	326 (85.6)	315 (82.7)	641	1.189	0.276
	Yes	55 (14.4)	66 (17.3)	121		
Gestational hypertension	No	370 (97.1)	366 (96.1)	736	0.637	0.425
	Yes	11 (2.9)	15 (3.9)	26		
Maternal smoking during pregnancy	Never smoked	372 (97.6)	376 (98.7)	748	1.164	0.281
	Smoked	9 (2.4)	5 (1.3)	14		
Maternal alcohol consumption during pregnancy	Never drank	371 (97.4)	380 (99.7)	751	-	0.011*
	Drank	10 (2.6)	1 (0.3)	11		
Pre-pregnancyBMI(kg/m^2^)	<18.5	15 (4.0)	5 (1.3)	20		
	18.5–23.9	289 (75.9)	328 (86.1)	617	15.013	0.002
	24–27.9	57 (14.9)	39 (10.2)	96		
	≥28	20 (5.2)	9 (2.4)	29		
Gestational weight gain (kg)	Inadequate	31 (8.1)	7 (1.8)	38		
	Appropriate	248 (65.1)	300 (78.7)	548	24.547	<0.001
	Excessive	102 (26.8)	74 (19.5)	176		
Paternal age (years)	<25	10 (2.6)	2 (0.5)	12		
	25–29	137 (36)	140 (36.8)	277	-	0.103*
	30–34	168 (44.1)	180 (47.2)	348		
	≥35	66 (17.3)	59 (15.5)	125		
Maternal age (years)	<25	19 (5.0)	13 (3.4)	32		
	25–29	154 (40.4)	167 (43.8)	321	2.228	0.526
	30–34	162 (42.5)	162 (42.5)	324		
	≥35	46 (12.1)	39 (10.3)	85		
Primary caregiver	Parents	144 (37.8)	189 (49.6)	333		
	Grandparents	228 (59.8)	184 (48.3)	412	10.839	0.004
	Nanny	9 (2.4)	8 (2.1)	17		
Family structure	Nuclear family	89 (23.4)	82 (21.5)	171	0.369	0.543
	Extended family	292 (76.6)	299 (78.5)	591		
Paternal education	Junior high school or below	9 (2.4)	1 (0.2)	10	-	<0.001*
	Senior high school	78 (20.5)	46 (12.1)	124		
	College or bachelor's degree	280 (73.5)	301 (79)	581		
	Master's degree or above	14 (3.6)	33 (8.7)	47		
Maternal education	Junior high school or below	5 (1.3)	3 (0.8)	8	-	0.224*
	Senior high school	16 (4.2)	20 (5.3)	38		
	College or bachelor's degree	319 (83.7)	300 (78.7)	619		
	Master's degree or above	41 (10.8)	58 (15.2)	97		
Paternal occupation	Mainly mental work	239 (62.7)	241 (63.3)	480	-	0.955*
	Mainly manual work	140 (36.8)	139 (36.5)	279		
	Unemployed	2 (0.5)	1 (0.2)	3		
Maternal occupation	Mainly mental work	295 (77.4)	300 (78.7)	595	5.198	0.074
	Mainly manual work	35 (9.2)	47 (12.3)	82		
	Unemployed	51 (13.4)	34 (9.0)	85		
Type of residence	Urban	329 (86.4)	322 (84.5)	651	1.311	0.519
	Suburban	47 (12.3)	50 (13.1)	97		
	Rural	5 (1.3)	9 (2.4)	14		
Annual household income (CNY)	≤80,000	22 (5.8)	14 (3.7)	36		
	80,001–150,000	186 (48.8)	173 (45.4)	359	10.895	0.012
	150,001–300,000	156 (40.9)	155 (40.7)	311		
	>300,000	17 (4.5)	39 (10.2)	56		

* Fisher's exact test was used for *P* values marked with an asterisk.

### 3.3 Multivariate analysis

Variables significant in the univariate analysis (*P* < 0.05) were entered into multivariate logistic regression. The results indicated that cesarean delivery (OR = 1.42, 95% CI: 1.05–1.93), maternal alcohol use during pregnancy (OR = 9.12, 95% CI: 1.09–76.33), pre-pregnancy overweight (OR = 1.62, 95% CI: 1.02–2.58) and obesity (OR = 2.54, 95% CI: 1.08–5.95), inadequate (OR = 4.09, 95% CI: 1.74–9.62) or excessive (OR = 1.59, 95% CI: 1.10–2.29) gestational weight gain, and grandparental caregiving (OR = 1.80, 95% CI: 1.32–2.46) were independently associated with an increased risk of cognitive developmental delay. Conversely, paternal education at the postgraduate level or above (OR = 0.06, 95% CI: 0.01–0.56) and an annual household income exceeding 300,000 CNY (OR = 0.31, 95% CI: 0.12–0.80) were associated with a decreased risk of cognitive developmental delay (Table 2).

**Table 2:** Multivariate logistic regression of factors associated with the risk of cognitive developmental delay in 12-month-old children.

Variables	*β*	S.E	Z	P	OR (95%CI)
Mode of delivery					
Vaginal					1.00 (Reference)
Cesarean delivery	0.35	0.16	2.28	0.023	1.42 (1.05–1.93)
Maternal alcohol use during pregnancy					
Never drank					1.00 (Reference)
Drank	2.21	1.08	2.04	0.042	9.12 (1.09–76.33)
Pre-pregnancy BMI (kg/m^2^)					
18.5–23.9					1.00 (Reference)
<18.5	1.07	0.55	1.94	0.053	2.91 (0.99–8.56)
24–27.9	0.48	0.24	2.04	0.041	1.62 (1.02–2.58)
≥28	0.93	0.43	2.14	0.032	2.54 (1.08–5.95)
Gestational weight gain					
Appropriate					1.00 (Reference)
Inadequate	1.41	0.44	3.22	0.001	4.09 (1.74–9.62)
Excessive	0.46	0.19	2.48	0.013	1.59 (1.10–2.29)
Primary caregiver					
Parents					1.00 (Reference)
Grandparents	0.59	0.16	3.72	<0.001	1.80 (1.32–2.46)
Nanny	0.72	0.54	1.34	0.181	2.05 (0.72–5.86)
Paternal education					
Junior high school or below					1.00 (Reference)
Senior high school	−1.56	1.10	−1.42	0.155	0.21 (0.02–1.80)
College or bachelor's degree	−2.08	1.09	−1.91	0.056	0.12 (0.01–1.05)
Master's degree or above	−2.79	1.13	−2.47	0.014	0.06 (0.01–0.56)
Annual household income (CNY)					
≤80,000					1.00 (Reference)
80,001–150,000	−0.39	0.38	−1.02	0.309	0.68 (0.32–1.43)
150,001–300,000	−0.45	0.38	−1.17	0.242	0.64 (0.30–1.35)
>300,000	−1.15	0.48	−2.41	0.015	0.31 (0.12–0.80)

## 4 Discussion

This case–control study strictly limited participants to full-term, singleton children without birth asphyxia or severe physical or neurological disorders. After controlling for known confounding factors, the study systematically analyzed determinants associated with the risk of cognitive developmental delay. Multivariate logistic regression analysis revealed that cesarean delivery, pre-pregnancy overweight and obesity, inadequate or excessive gestational weight gain, maternal alcohol consumption during pregnancy, and grandparental primary caregiving were associated with elevated risk of cognitive developmental delay in children, whereas higher paternal education level and higher annual household income were independent protective factors for lower cognitive developmental delay risk.

After adjusting for other confounders, cesarean delivery remained associated with a 42% higher risk of cognitive developmental delay (OR = 1.42), revealing a potential independent association between delivery mode and early cognitive developmental outcomes. Several domestic and international studies have reported findings consistent with the present results. Polidano et al. observed that children delivered by cesarean delivery exhibited poorer vocabulary, reading, and writing abilities between ages 4 and 9 compared to those born vaginally (14). Similarly, studies conducted in China reported that cesarean-born infants generally showed lower levels of intellectual development than those born by vaginal delivery (15, 16). Previous research has suggested that this difference may be closely related to alterations in early microbial colonization patterns. During vaginal delivery, neonates are exposed to diverse maternal microbial communities from the vagina, gut, and skin—such as Lactobacillus, Prevotella, and Klebsiella—while cesarean delivery disrupts vertical transmission of maternal microbiota, leading to colonization mainly from maternal skin and hospital environments (17). Accumulating evidence indicates that the gut microbiota, an essential component of human health, plays a critical role in immune maturation, metabolic regulation, and central nervous system development. It can modulate neural function through the gut–brain axis (GBA), transmitting signals via immune pathways and the vagus nerve, regulating tryptophan metabolism, promoting gut hormone secretion, and producing neurotransmitters (e.g., GABA) and short-chain fatty acids (SCFAs) that affect cognition and behavior (18). A recent study has further shown that vaginal microbiota transfer in cesarean-born infants can partially restore gut microbial composition and may enhance neurodevelopment by modulating specific bacterial taxa and metabolites (19).

This study also found that both inadequate and excessive gestational weight gain were significantly associated with children's cognitive developmental risk. Deviation from the recommended gestational weight gain range may adversely affect fetal brain development, as suggested by existing physiological and epidemiological evidence (20). Optimal maternal weight gain during pregnancy can guarantee sufficient fetal nutrition, maintain normal placental perfusion, and support neurological maturation. Consistent with our findings, multiple epidemiological studies have also reported a link between abnormal gestational weight gain and offspring cognitive development (21–23). Mechanistically, existing literature has proposed several potential biological pathways to explain this association. Excessive gestational weight gain and the resulting accumulation of maternal and fetal adipose tissue may induce chronic low-grade inflammation, accompanied by elevated proinflammatory cytokines such as interleukin-6 and tumor necrosis factor-α. Such an inflammatory microenvironment is considered to interfere with normal central nervous system development (24). At the molecular level, persistent inflammation can alter proinflammatory signaling, exacerbate lipotoxicity and oxidative stress, and impair insulin, glucose, leptin, and IGF pathways, resulting in mild insulin resistance and impaired receptor signaling in the hypothalamus and hippocampus, which ultimately affects neurogenesis, synaptic plasticity, and neural network formation (25, 26). Moreover, gut microbial dysbiosis may mediate the relationship between abnormal gestational weight gain and neurodevelopmental outcomes. Mothers with excessive weight gain have been shown to possess lower gut microbiota diversity, and maternal microbial profiles are strongly correlated with those of their offspring (27). Thus, excessive gestational weight gain may alter maternal and fetal gut microbiota, activate immune responses, and increase the risk of neurodevelopmental disorders. Conversely, insufficient weight gain may reflect maternal undernutrition, leading to inadequate fetal nutrient supply and impaired brain development (28). Therefore, maintaining appropriate gestational weight gain is crucial for supporting fetal neural development and reducing cognitive developmental risk.

This study further found that grandparental primary caregiving, maternal alcohol consumption during pregnancy, and pre-pregnancy obesity were associated with a higher risk of cognitive developmental delay. In contrast, higher paternal educational level and annual household income were linked to a lower risk. Paternal education is strongly linked to children's cognitive outcomes; fathers with higher education levels tend to possess better parenting knowledge and more positive attitudes, providing greater cognitive stimulation and emotional support (29, 30). In contrast, fathers with limited education may show insufficient involvement in early learning, reduced parent–child interaction, and a lack of awareness regarding early developmental needs, thereby constraining cognitive progress. Children primarily cared for by grandparents were at higher risk of cognitive developmental delay, which may be attributed to several factors. Grandparents may have more traditional caregiving beliefs and limited awareness of evidence-based parenting and early education (31). In addition, they may have lower levels of educational attainment, which could limit their ability to provide cognitively stimulating activities. Age-related physical limitations may also reduce the frequency and quality of interactive play and responsive caregiving (32). Parents, on the other hand, are more likely to engage in cognitively enriching activities such as guided reading, interactive play, and participation in early education programs, all of which are beneficial for cognitive enhancement (33). Previous research has likewise confirmed that increased parental companionship and parent–child interaction significantly contribute to improvements in children's cognitive performance (34). In this context, although parental education and household income were used as indicators of socioeconomic status, these variables may not fully reflect the immediate caregiving environment when grandparents serve as primary caregivers. Family economic status emerged as another critical determinant. Studies have shown that in low- and middle-income countries, approximately 43% of children under five fail to achieve their developmental potential due to poverty. Low household income may adversely influence cognitive development through several mechanisms, including limited access to educational resources, restricted learning environments, and elevated family stress (35). Further evidence suggests that strengthening parental support and improving the quality of caregiving and education in low-income families can partially offset the detrimental effects of poverty on children's cognitive development (36). Moreover, both maternal alcohol consumption during pregnancy and pre-pregnancy obesity were associated with an increased risk of cognitive developmental delay. However, the association with maternal alcohol consumption should be interpreted with caution. In the present study, the number of mothers reporting alcohol consumption during pregnancy was very small, which resulted in a wide confidence interval and limited precision of the effect estimate. Therefore, the magnitude of this association may be unstable and potentially overestimated. Previous research has demonstrated that maternal alcohol consumption during pregnancy can disrupt the structural and functional development of the fetal brain; even low levels of alcohol intake may lead to white matter abnormalities and behavioral impairments in children (37, 38). As reported in existing studies, pre-pregnancy overweight and obesity may alter maternal metabolic and hormonal pathways, adversely affecting fetal neurodevelopment and subsequently reducing children's cognitive and socio-emotional functioning (39).

No significant associations were observed between maternal age, maternal education, or infant feeding method and cognitive outcomes, which may reflect the relatively high educational level and regional homogeneity of the study population. Overall, the findings suggest that both maternal health and family social environment jointly influence early cognitive development. Comprehensive interventions targeting high-risk populations—such as gestational weight management, alcohol use screening, and evidence-based parenting—are warranted to reduce the risk of cognitive developmental delay.

## 5 Conclusion

This study found that cesarean delivery, maternal alcohol use during pregnancy, pre-pregnancy overweight and obesity, abnormal gestational weight gain, and grandparental caregiving were associated with an increased risk of cognitive developmental delay in 12-month-old children, whereas higher paternal education and higher annual household income were associated with a reduced risk. These findings highlight the combined influence of maternal health, perinatal conditions, caregiving arrangements, and socioeconomic context on early cognitive development. The results underscore the importance of integrating modifiable perinatal factors, including delivery mode and maternal weight-related indicators, with caregiving and socioeconomic information in early developmental risk screening. Strengthening prenatal health management, promoting appropriate weight control, implementing alcohol-use screening during pregnancy, and enhancing family-based parenting education may help reduce the risk of cognitive developmental delay and provide practical evidence for future targeted intervention studies.

## 6 Limitations

Several limitations should be noted. First, as a case-control study, causal inferences are limited. Second, all participants were recruited from Sichuan Provincial Maternity and Child Health Care Hospital in the central urban area of Chengdu. Although this sample adequately represents the local urban population, it underrepresents rural populations, low-income families, and less educated groups outside central Chengdu. In addition, the predominantly urban study sample may be subject to unmeasured environmental exposures unique to urban settings, such as air pollution, noise, and psychosocial stress, which were not assessed in this study and may act as unmeasured confounders for cognitive development. Third, the DDST is a screening tool rather than a diagnostic instrument; by combining the “suspected” and “abnormal” categories, we aimed to improve sensitivity for early risk identification, but this approach may have overestimated the true prevalence of developmental delay in the absence of subsequent confirmatory diagnostic assessment. Although the DDST covers multiple developmental domains, this case–control study only adopted its overall screening classification. Complete item and domain-level data were unavailable. Further subdomain analyses could not be performed, especially for the language domain. Future studies should use detailed domain-specific DDST results together with standardized language and cognitive assessments. Fourth, although major confounders were statistically controlled, residual confounding from unmeasured variables cannot be excluded. Maternal infections, consanguinity, environmental toxin exposure, and subclinical or later-onset maternal or child hypothyroidism were not specifically assessed due to data limitations. These factors have been reported to influence neurodevelopment and should be considered in future studies. Fifth, the prevalence of certain exposures in our sample was very low, particularly maternal alcohol consumption during pregnancy. The small number of exposed cases may have reduced the statistical power and resulted in unstable effect estimates with wide confidence intervals. Therefore, the corresponding findings should be interpreted with caution. Sixth, cognitive development was assessed using cross-sectional data, which limits the ability to capture long-term developmental trajectories. In addition, information on socioeconomic status was limited to parental characteristics, which may not fully capture the caregiving environment in families where grandparents are the primary caregivers.

## Data Availability

The raw data supporting the conclusions of this article will be made available by the authors, without undue reservation.
